# Management of diabetes mellitus patients with sickle cell anemia: Challenges and therapeutic approaches

**DOI:** 10.1097/MD.0000000000037941

**Published:** 2024-04-26

**Authors:** Emmanuel Ifeanyi Obeagu, Getrude Uzoma Obeagu

**Affiliations:** a Department of Medical Laboratory Science, Kampala International University, Kampala, Uganda; b School of Nursing Science, Kampala International University, Kampala, Uganda.

**Keywords:** diabetes mellitus, glycemic control, multidisciplinary care, pain management, sickle cell anemia, therapeutic approaches

## Abstract

The coexistence of diabetes mellitus (DM) and sickle cell anemia (SCA) poses significant challenges in clinical management due to the complex interactions and overlapping complications associated with both conditions. Managing diabetes in individuals with SCA requires a comprehensive approach that addresses the unique physiological and pathological aspects of both diseases. This paper reviews the challenges encountered in the management of DM in patients with SCA and explores therapeutic strategies and approaches to optimize patient care. Challenges in the management of DM in individuals with SCA stem from several factors, including the impact of hemoglobin variants on glycemic control assessment, increased susceptibility to infections, altered immune response, and complications associated with both diseases. Moreover, the coexistence of SCA and DM heightens the susceptibility to infections due to compromised immune function, emphasizing the need for vigilant preventive measures, including vaccinations and close monitoring for infectious complications. Close collaboration among healthcare providers specializing in diabetes, hematology, and other relevant fields is crucial for developing comprehensive care plans. Individualized treatment strategies that balance glycemic control, pain management, and preventive care are essential to mitigate complications and optimize the overall health outcomes of patients with both DM and SCA. In conclusion, managing diabetes in the context of SCA necessitates a nuanced and patient-centered approach. By addressing the challenges and employing tailored therapeutic strategies, healthcare providers can improve the quality of life and health outcomes for individuals affected by both conditions.

## 1. Introduction

Diabetes mellitus (DM) and sickle cell anemia (SCA) are 2 distinct, chronic conditions that individually present considerable challenges to patients and healthcare providers.^[1]^ DM, characterized by persistent hyperglycemia, impacts millions of lives worldwide.^[2]^ SCA, a hereditary hemoglobinopathy, leads to the formation of abnormal, rigid red blood cells and is associated with acute and chronic complications. However, when both conditions coexist within a single individual, the complexities and risks are compounded, creating a medical confluence often referred to as “double trouble.^[3–7]^ The management of DM in SCA patients requires a specialized and nuanced approach.^[8]^ The interplay between the 2 conditions introduces a unique set of challenges that encompass glycemic control, hemolytic crises, chronic pain, end-organ damage, and an increased susceptibility to infections.^[9]^ To provide the best possible care for these patients, healthcare providers must understand the intricacies of managing DM within the context of SCA.^[10]^

This paper seeks to explore the challenges faced by individuals living with both DM and SCA, shedding light on the complex interrelationship between these conditions. It will also discuss therapeutic approaches and strategies that can be employed to optimize the management of DM in SCA patients, reduce complications, and ultimately enhance their quality of life. As we embark on this journey through the intersection of 2 chronic conditions, our aim is to provide insights and guidance for healthcare providers, researchers, and patients grappling with the complexities of “double trouble.”

## 2. Prevalence of DM among people with SCA and SCA among people with DM

Limited data exist on the prevalence of DM specifically among individuals with SCA. However, individuals with SCA are at risk for various complications, including endocrine disorders like diabetes, due to chronic inflammation, oxidative stress, and other factors associated with the disease. Some studies suggest that the prevalence of diabetes among individuals with SCA may be higher than in the general population due to these factors, but more research is needed to establish a clear prevalence rate.^[9]^ Similarly, data on the prevalence of SCA among individuals with DM are limited. However, individuals with DM may have an increased risk of various comorbidities, including hematologic disorders like SCA, due to factors such as chronic inflammation, oxidative stress, and vascular complications associated with diabetes. Studies examining the prevalence of SCA among individuals with DM are sparse, but given the increased awareness of diabetes-related complications, there may be increased recognition of SCA among individuals with DM in clinical practice.^[10]^

## 3. Diabetes mellitus in SCA

The coexistence of diabetes mellitus (DM) and SCA presents a complex and challenging medical scenario.^[11]^ DM is a metabolic disorder characterized by elevated blood glucose levels due to insufficient insulin production or insulin resistance.^[12–15]^ SCA, on the other hand, is a hereditary hemoglobinopathy that results in the formation of abnormal, crescent-shaped red blood cells, which can lead to a range of complications.^[16]^

## 4. Pathophysiological mechanisms linking SCA to DM

The pathophysiological mechanisms linking SCA to diabetes mellitus (DM) are complex and multifactorial. Both SCA and DM involve alterations in cellular and molecular pathways that can contribute to the development of complications. SCA is characterized by chronic inflammation and oxidative stress due to the hemolytic nature of the disease. Persistent inflammation and oxidative stress can contribute to insulin resistance and impair pancreatic beta-cell function, leading to the development of diabetes. Both SCA and DM are associated with endothelial dysfunction, which can lead to impaired blood flow and microvascular complications. Endothelial dysfunction in diabetes is well-known, and in SCA, vaso-occlusion and ischemia-reperfusion injury contribute to endothelial dysfunction. This shared vascular pathology may exacerbate the risk of complications, including diabetic vascular complications.^[7]^

SCA has been associated with insulin resistance, a condition in which cells respond less effectively to insulin. Insulin resistance is a key factor in the development of type 2 diabetes. The chronic inflammatory state in SCA, along with potential alterations in adipokines and cytokines, may contribute to insulin resistance. SCA may impact pancreatic beta-cell function, leading to impaired insulin secretion. Chronic inflammation and oxidative stress in SCA may contribute to pancreatic beta-cell damage and dysfunction, increasing the risk of developing diabetes. Both SCA and DM have genetic components. Genetic factors influencing the risk of SCA or related complications may also play a role in the development of diabetes. Additionally, genetic factors contributing to diabetes susceptibility may interact with those associated with SCA. SCA is characterized by vaso-occlusion, leading to tissue hypoxia and ischemia-reperfusion injury. These processes may contribute to the development of insulin resistance and damage to insulin-sensitive tissues. Complications associated with SCA, such as renal dysfunction and hypertension, may contribute to the development of diabetes. Additionally, medications used in the management of SCA, such as hydroxyurea, may have metabolic effects that can influence glucose metabolism.^[9]^

## 5. Why people with SCA and DM have increased risk of hypoglycemia in comparison with people with DM without SCA

Individuals with SCA and diabetes mellitus (DM) may have an increased risk of hypoglycemia compared to those with DM alone due to several interconnected factors. The interaction between these 2 conditions can create a complex health landscape, leading to heightened vulnerability to low blood glucose levels. SCA can affect the normal counterregulatory response to hypoglycemia. The chronic inflammation and oxidative stress associated with SCA may impair the body’s ability to respond adequately to low blood glucose levels, making it challenging to correct hypoglycemia effectively. Both SCA and DM are characterized by chronic inflammation and oxidative stress. This systemic inflammation can contribute to insulin resistance and impair the body’s ability to regulate glucose effectively.^[9]^

In the presence of diabetes, this can lead to a heightened risk of hypoglycemia, especially if insulin or other glucose-lowering medications are being used. Individuals with SCA may be on medications such as hydroxyurea, which can affect glucose metabolism and increase the risk of hypoglycemia when combined with diabetes medications. Additionally, managing SCA and DM concurrently may require a delicate balance of medications, making it more challenging to avoid hypoglycemic episodes. SCA can lead to organ dysfunction, including renal impairment. Individuals with compromised kidney function may have altered clearance of medications, affecting the metabolism of glucose-lowering drugs and increasing the risk of hypoglycemia. SCA has been associated with altered glucose metabolism, including insulin resistance. The presence of insulin resistance in individuals with diabetes can lead to difficulties in maintaining optimal blood glucose levels, contributing to an increased risk of hypoglycemia. The vaso-occlusive crises associated with SCA can result in tissue hypoxia and ischemia-reperfusion injury. These events may exacerbate insulin resistance and contribute to fluctuations in blood glucose levels, increasing the likelihood of hypoglycemia.^[10]^

## 6. Challenges in managing DM in SCA patients

### 6.1. Hemoglobin A1c (HbA1c) interpretation of DM in SCA

Interpreting Hemoglobin A1c (HbA1c) levels in individuals with both diabetes mellitus (DM) and SCA can be challenging due to the presence of hemoglobin variants inherent in SCA.^[17]^ HbA1c is a test used to measure average blood sugar levels over the past 2 to 3 months by assessing the amount of glucose that has attached to hemoglobin in red blood cells.^[17]^ However, in SCA, the presence of abnormal hemoglobin variants, particularly hemoglobin S (HbS), can interfere with the accuracy of HbA1c measurements.^[18]^ Hemoglobin variants present in SCA can lead to erroneous or misleading HbA1c results, potentially either underestimating or overestimating the actual average blood sugar levels.^[19]^ The standard HbA1c test assumes a typical lifespan of red blood cells (around 120 days).^[20]^ But in individuals with SCA, red blood cells can have a shorter lifespan due to hemolysis (destruction of red blood cells), resulting in a turnover of red blood cells more frequently than every 120 days.^[21]^ This accelerated red blood cell turnover can affect the accuracy of HbA1c measurements.^[22]^ Therefore, the interpretation of HbA1c levels in individuals with both DM and SCA requires caution.^[23]^ Healthcare providers should consider alternative methods for assessing glycemic control in these individuals. Some alternatives include: Regular self-monitoring of blood glucose levels using a glucometer to track day-to-day variations in blood sugar levels. Fructosamine is a measure of glycated serum proteins (not affected by hemoglobin variants) and reflects shorter-term glucose control over a few weeks.^[24]^ It can serve as an alternative marker in cases where HbA1c might be unreliable. Continuous Glucose Monitoring (CGM) devices provide continuous monitoring of interstitial glucose levels, offering a more comprehensive picture of glucose fluctuations throughout the day.^[25]^ Consideration of clinical symptoms, patient history, and other tests to evaluate glycemic control and the effectiveness of diabetes management in individuals with SCA and DM.^[26]^ Healthcare providers managing diabetes in individuals with SCA should be aware of these limitations and consider alternative methods to monitor and assess glycemic control accurately. Tailored approaches that consider the challenges posed by the presence of hemoglobin variants in SCA are essential for appropriate diabetes management in this population. Consulting with specialists familiar with both conditions can aid in selecting the most suitable methods for monitoring and managing diabetes in individuals with SCA.

### 6.2. Increased risk of hypoglycemia of DM in SCA

The interplay between diabetes mellitus (DM) and SCA can contribute to an increased risk of hypoglycemia (low blood sugar) in individuals affected by both conditions.^[27]^ Managing diabetes in the presence of sickle cell disease requires careful attention due to various factors that can heighten the susceptibility to hypoglycemia.^[28]^ Hemoglobin variants, such as hemoglobin S (HbS) in SCA, can interfere with accurate glucose monitoring, including methods like Hemoglobin A1c (HbA1c) and fingerstick blood glucose tests.^[29]^ These variants can lead to misleading glucose readings, potentially causing difficulties in regulating insulin or other diabetes medications, thereby increasing the risk of hypoglycemia or hyperglycemia due to misinterpretation of blood sugar levels. SCA is characterized by vaso-occlusive crises, where blood flow is obstructed by sickled red blood cells.^[30,31]^ During these crises, the body’s metabolic demands can increase, potentially affecting glucose utilization and altering blood sugar levels. Such fluctuations might make it challenging to maintain stable blood glucose levels and increase the risk of hypoglycemia. Sickle cell disease can affect various organs, potentially impacting their function, including the pancreas, which produces insulin.^[32]^

Dysfunction of the pancreas due to SCA-related complications could influence insulin production and secretion, contributing to fluctuations in blood sugar levels. Some medications used in the management of sickle cell disease may interact with diabetes medications or affect their absorption, potentially influencing blood glucose levels and increasing the risk of hypoglycemia.^[33]^ Given these complexities, individuals with both DM and SCA require specialized and cautious management to minimize the risk of hypoglycemia.^[34]^ Frequent monitoring of blood glucose levels using methods less affected by hemoglobin variants, such as continuous glucose monitoring (CGM) or fructosamine levels.^[35]^

Tailoring diabetes management plans to accommodate the challenges posed by SCA and considering factors like the patient’s overall health status, organ function, and potential medication interactions. Close collaboration between healthcare providers specializing in diabetes and sickle cell disease to develop a comprehensive care plan that addresses the unique needs and challenges of managing both conditions. Careful consideration and close monitoring are essential to managing diabetes effectively in individuals with SCA, minimizing the risk of hypoglycemia, and ensuring overall health and well-being.

Some medications commonly used in the management of sickle cell disease may include hydroxyurea, pain relievers (such as opioids), and antibiotics. While these medications primarily target sickle cell disease, potential interactions with diabetes medications could occur. For example, opioids can sometimes cause constipation, which may impact the absorption or effectiveness of certain diabetes medications. It is crucial for individuals with both sickle cell disease and diabetes to communicate openly with their healthcare providers about all medications they are taking. Healthcare professionals can assess potential interactions, monitor for side effects, and make adjustments to the treatment plan if necessary. Patients should not make any changes to their medication regimen without consulting their healthcare team. It is advisable to consult with a healthcare professional or pharmacist for the most up-to-date and personalized advice based on the specific medications and health conditions involved.^[35]^

### 6.3. Chronic pain and pain medications of DM in SCA

Chronic pain management in individuals with both diabetes mellitus (DM) and SCA presents a unique challenge due to the coexistence of 2 conditions that can independently cause pain and complications.^[21]^ Managing chronic pain in these individuals requires a careful approach considering the specific needs and potential interactions between treatments for diabetes and sickle cell disease. Both DM and SCA can cause chronic pain, but the nature and characteristics of pain can differ.^[36]^ SCA is known for vaso-occlusive crises, leading to acute, severe, and intermittent pain episodes, while individuals with diabetes may experience neuropathic pain, often described as burning, tingling, or shooting pain due to nerve damage. When managing pain in individuals with both conditions, healthcare providers need to consider potential interactions between pain medications and medications used to manage diabetes or sickle cell disease.^[37]^ For instance, some pain medications, particularly opioids, may affect blood sugar levels or interact with medications used in diabetes management. Developing an individualized pain management plan is crucial. This plan should consider the type and severity of pain, the impact on daily functioning, potential side effects of medications, and any complications associated with diabetes or sickle cell disease.^[38]^ Integrating nonpharmacological approaches for pain management is essential.^[39]^ Techniques such as physical therapy, exercise, cognitive-behavioral therapy (CBT), relaxation techniques, acupuncture, and mindfulness-based practices can help alleviate pain and improve quality of life without relying solely on medications.^[40]^ Collaborative care involving healthcare providers specializing in pain management, diabetes, and sickle cell disease is important.^[41]^ This interdisciplinary approach ensures comprehensive care, considering the complexities of both conditions and tailoring treatments accordingly. Given the potential risks of opioid medications, including addiction, tolerance, and adverse effects, their use in managing chronic pain should be carefully considered and monitored closely. Healthcare providers might opt for alternative medications or limit the use of opioids to specific situations where they are deemed necessary and when the benefits outweigh the risks. Individuals with DM and SCA require a personalized approach to chronic pain management that considers the complexities of both conditions.^[42]^ Balancing pain relief with the management of diabetes and sickle cell disease is crucial to ensure optimal outcomes while minimizing the risks associated with medications and potential interactions. Regular follow-ups, ongoing assessments, and open communication between patients and healthcare providers are essential components of effective chronic pain management in this population.

### 6.4. End-organ damage of DM in SCA

Diabetes mellitus (DM) and SCA are both chronic conditions that can independently lead to end-organ damage, and when occurring together, they may heighten the risk and severity of complications affecting various organs in the body.^[43]^ Diabetes significantly increases the risk of cardiovascular complications.^[44]^ Individuals with diabetes have an elevated risk of atherosclerosis, coronary artery disease, heart attacks, strokes, and peripheral vascular disease due to damage to blood vessels from high blood sugar levels. Diabetes is a leading cause of chronic kidney disease (diabetic nephropathy) characterized by damage to the small blood vessels in the kidneys.^[45]^ This damage can progress to kidney failure, requiring dialysis or transplantation. Diabetic retinopathy is a complication that affects the eyes, leading to damage to the blood vessels of the retina. Left untreated, it can result in vision impairment and even blindness.^[46]^ Diabetic neuropathy is nerve damage caused by diabetes, leading to symptoms such as numbness, tingling, pain, or weakness, primarily affecting the feet and hands.^[47]^ SCA can lead to functional asplenia due to repeated episodes of splenic infarction (blockage of blood flow), increasing the risk of infections, particularly by encapsulated bacteria.^[48]^ Vaso-occlusive crises in SCA cause severe, episodic pain due to the blockage of blood vessels by sickled red blood cells. These crises can result in damage to various organs, contributing to chronic complications.^[49–51]^ Vaso-occlusion can affect multiple organs, including the lungs (acute chest syndrome), bones, liver, and brain, leading to acute and chronic complications.^[49]^ When DM and SCA coexist, individuals may experience an increased risk and severity of complications affecting different organs. The combination of these conditions can further exacerbate the risk of end-organ damage due to the cumulative effects of both diseases on blood vessels, organ function, and overall health.^[52]^ Managing both DM and SCA involves a comprehensive approach aimed at preventing complications, preserving organ function, and improving quality of life. This may include regular medical checkups, monitoring blood sugar levels and hemoglobin, adhering to treatment plans, maintaining a healthy lifestyle, managing complications promptly, and seeking specialized care from healthcare providers experienced in managing both conditions. Collaborative and multidisciplinary care is essential for effectively addressing the complexities and reducing the risk of end-organ damage in individuals with DM and SCA.

### 6.5. Infection susceptibility of DM in SCA

The coexistence of diabetes mellitus (DM) and SCA can potentially impact the immune system, increasing the susceptibility to infections.^[53]^ Both DM and SCA can independently compromise the immune response, which may lead to an increased risk of certain types of infections when both conditions are present.^[54]^ High blood sugar levels in diabetes can weaken the immune system’s ability to fight infections.^[55]^ Hyperglycemia affects various aspects of immune function, impairing the response of white blood cells (such as neutrophils and macrophages) that play a crucial role in defending the body against pathogens.^[56]^ Individuals with diabetes are at an increased risk of infections, such as urinary tract infections, skin infections (e.g., cellulitis), fungal infections (e.g., candidiasis), and respiratory infections. SCA is associated with functional asplenia or hyposplenism, where the spleen’s function is compromised due to repeated infarctions and damage caused by sickled red blood cells.^[57]^ As a result, individuals with SCA, especially children, have an increased susceptibility to infections, particularly those caused by encapsulated bacteria like Streptococcus pneumoniae and Haemophilus influenzae.^[58]^ This susceptibility to bacterial infections is due to the spleen’s role in fighting infections, and the absence or impaired function of the spleen increases the risk of certain bacterial infections. When diabetes and SCA coexist, there may be an additive effect on the susceptibility to infections due to the compromised immune responses associated with both conditions.^[59]^ This combination can potentially increase the risk and severity of infections caused by various pathogens, including bacteria, viruses, and fungi. Ensuring appropriate vaccinations, especially against bacterial infections like pneumococcus and meningococcus, to prevent potential infections in individuals with SCA and diabetes.^[60]^ Close monitoring of blood sugar levels in diabetes and prompt management of hyperglycemia to support immune function.^[61]^ In some cases, healthcare providers might recommend antibiotic prophylaxis to prevent specific infections, especially in individuals with SCA and functional asplenia.^[62]^ Emphasizing good hygiene practices, a balanced diet, adequate rest, stress reduction, and avoidance of harmful habits like smoking to support immune function in individuals with both conditions.^[63]^ Consulting healthcare providers specializing in both diabetes and SCA is crucial for developing a comprehensive plan to manage these conditions while minimizing the risk of infections.^[64]^ Regular follow-ups and proactive measures are essential for optimizing the health and well-being of individuals affected by both DM and SCA.^[65]^

## 7. Therapeutic approaches

### 7.1. Multidisciplinary care of DM in SCA

Multidisciplinary care for individuals affected by both diabetes mellitus (DM) and sickle cell anemia (SCA) involves a coordinated approach by a team of healthcare professionals from various specialties.^[64]^ This comprehensive approach aims to address the complexities and unique needs of managing both conditions simultaneously. Managing DM in the presence of SCA requires careful coordination among healthcare providers to optimize patient care and prevent complications.^[65]^ Here are the key components of multidisciplinary care for DM in individuals with SCA: A team of healthcare professionals specializing in diabetes management, sickle cell disease, and related fields (hematology, endocrinology, primary care, etc) work collaboratively to develop and implement an integrated care plan.^[64]^ Scheduled regular checkups are crucial to monitor both conditions, including blood sugar levels, HbA1c, hemoglobin levels, organ function, and potential complications associated with DM and SCA.^[63]^ Tailoring treatment plans to accommodate the unique challenges posed by the coexistence of DM and SCA.^[66]^ This involves managing blood sugar levels, preventing and managing sickle cell crises, and addressing complications specific to each condition. Careful selection and monitoring of medications used in the management of diabetes and sickle cell disease to minimize potential interactions and optimize treatment outcomes.^[63]^ Implementing appropriate pain management strategies considering the complexities of chronic pain in individuals with both DM and SCA.^[67]^ This may involve nonpharmacological approaches, analgesics, and other medications tailored to the individual’s needs. Providing education and support to empower patients and caregivers in managing both conditions effectively.^[68]^ This includes guidance on self-care, symptom recognition, medication adherence, lifestyle modifications, and when to seek medical attention. Addressing the emotional and psychological aspects of living with both chronic conditions by providing counseling, support groups, and resources to help individuals cope with the challenges. Emphasizing preventive care measures, such as vaccinations, screenings, healthy lifestyle modifications, and disease management strategies to prevent complications and promote overall health.^[69]^ Effective multidisciplinary care for individuals with both DM and SCA requires close collaboration, communication, and coordination among healthcare providers and the patient.^[70]^ Tailoring the care plan to address the unique needs and challenges of managing these coexisting conditions is essential for optimizing health outcomes and improving the quality of life for affected individuals.

### 7.2. Individualized treatment plans of DM in SCA

Individualized treatment plans for managing diabetes mellitus (DM) in the context of sickle cell anemia (SCA) are essential due to the unique challenges posed by both conditions.^[71]^ The coexistence of DM and SCA requires a tailored approach that considers the complexities and potential interactions between treatments for each condition. Conduct a thorough assessment of the individual’s medical history, including the duration and severity of both DM and SCA, any complications, previous treatments, medications, and current symptoms.^[72]^ Involve a multidisciplinary team of healthcare professionals experienced in managing both DM and SCA.^[64]^ This team may include hematologists, endocrinologists, primary care physicians, diabetes educators, pain specialists, and other relevant specialists. Implement a schedule for regular monitoring of both conditions. This includes monitoring blood glucose levels, HbA1c, hemoglobin levels, organ function (especially kidney and liver function), and any complications associated with DM and SCA.^[72]^ Develop an individualized diabetes management plan that accounts for the challenges posed by SCA.^[73]^ This plan should focus on controlling blood sugar levels, considering the potential limitations in using standard diabetes monitoring methods like HbA1c due to the presence of hemoglobin variants in SCA. Address chronic pain associated with SCA while considering the impact of pain medications on blood sugar levels and potential interactions with diabetes medications.^[74]^ Implement tailored pain management strategies that balance pain relief with diabetes management goals. Carefully select medications for managing diabetes, taking into account their efficacy, potential side effects, and interactions with medications used in sickle cell disease.^[37]^ Monitor for any adverse effects or interactions that may affect either condition. Emphasize the importance of healthy lifestyle habits, including a balanced diet, regular physical activity, adequate hydration, stress management, and avoiding harmful habits like smoking or excessive alcohol consumption. Provide comprehensive education to the individual and caregivers regarding both conditions, including symptom recognition, medication adherence, self-monitoring, recognizing and managing sickle cell crises, and seeking timely medical attention. Implement preventive measures such as vaccinations (including pneumococcal and influenza vaccines), regular screenings, and health maintenance to prevent complications associated with both DM and SCA.^[75]^ Schedule regular follow-up appointments to assess treatment effectiveness, adjust management plans as needed, and ensure ongoing communication and collaboration between the healthcare team and the individual. Individualized treatment plans for managing DM in individuals with SCA should be flexible, regularly reviewed, and adapted based on the individual’s response to treatment, changing health status, and evolving needs.^[74]^ The aim is to optimize management strategies for both conditions while minimizing risks and complications. Close collaboration among healthcare providers and open communication with the individual are crucial for successful management.

### 7.3. Glycemic control strategies of DM in SCA

Managing glycemic control in individuals with both diabetes mellitus (DM) and sickle cell anemia (SCA) requires a tailored approach that addresses the challenges of monitoring blood sugar levels accurately and managing diabetes while considering the complexities of sickle cell disease.^[76]^ Due to the interference of hemoglobin variants in SCA with standard HbA1c measurements, consider alternative methods for assessing glycemic control.^[18]^ Develop individualized diabetes management plans that consider the challenges posed by SCA.^[77]^ Tailor treatment goals, medications, and lifestyle modifications to the specific needs and limitations of the individual. Select diabetes medications that are suitable for individuals with SCA and diabetes, considering their efficacy, safety profile, and potential interactions with medications used in sickle cell disease.^[78–80]^ Ensure close monitoring for any adverse effects or interactions. Emphasize the importance of a healthy diet and lifestyle modifications to manage blood sugar levels effectively. Encourage a balanced diet, regular physical activity, adequate hydration, stress management, and avoidance of harmful habits. Engage a multidisciplinary healthcare team, including specialists in diabetes, hematology, and other relevant fields, to coordinate care, provide comprehensive guidance, and address the unique challenges associated with managing both conditions.^[81]^ Provide extensive education to the individual and caregivers about managing both diabetes and sickle cell disease.^[82]^ Include guidance on self-monitoring, symptom recognition, medication adherence, recognizing sickle cell crises, and when to seek medical attention. Schedule regular follow-up visits to monitor the effectiveness of diabetes management strategies, make necessary adjustments, and ensure ongoing support and guidance for the individual. Implement preventive measures, such as vaccinations (e.g., pneumococcal and influenza vaccines), regular screenings, and health maintenance, to prevent complications associated with both DM and SCA.^[83]^ Managing glycemic control in individuals with both DM and SCA requires a personalized and vigilant approach that takes into account the challenges and complexities of managing both conditions simultaneously.^[84]^ Close collaboration among healthcare providers and active participation from the individual are crucial for optimizing glycemic control while managing the complexities of sickle cell disease. Table 1 Shows medications in Sickle Cell Anemia Patients with Diabetes

**Table 1:** Showing medications in sickle cell anemia patients with diabetes.

Medications for sickle cell disease	Potential interaction with diabetes medications
Hydroxyurea	May require dosage adjustments for diabetes medications; monitor blood glucose levels.
Opioid pain relievers	May cause constipation, potentially affecting the absorption or efficacy of certain diabetes medications. Adjustments may be needed.
Antibiotics	No direct interaction with diabetes medications, but consider the overall impact on health and potential side effects. Communicate with healthcare providers.

The choice of diabetes medications for individuals with sickle cell disease (SCD) and diabetes should be made based on a careful consideration of the efficacy, safety profile, and potential interactions with medications used in sickle cell disease. It is crucial for healthcare providers to tailor the treatment plan to the individual’s specific health needs.^[83,84]^ Here are some diabetes medications commonly used, along with considerations for individuals with sickle cell disease:

Metformin:

○ **Efficacy:** Effective in improving insulin sensitivity and lowering blood glucose levels.
○ **Safety Profile:** Generally well-tolerated, but may cause gastrointestinal side effects.
○ **Considerations:** No direct interactions with medications commonly used in sickle cell disease. Monitor for potential gastrointestinal side effects.

Insulin:

○ **Efficacy:** Can be highly effective in managing blood glucose levels.
○ **Safety Profile:** Generally safe when used appropriately.
○ **Considerations:** No specific interactions with sickle cell disease medications. Individualized dosing and monitoring are crucial.

SGLT-2 Inhibitors (e.g., canagliflozin, dapagliflozin):

○ **Efficacy:** Helps lower blood glucose levels by promoting glucose excretion in the urine.
○ **Safety Profile:** Can be associated with urinary tract infections and increased risk of genital mycotic infections.
○ **Considerations:** No significant interactions with sickle cell disease medications reported. Monitor for potential infections.

DPP-4 Inhibitors (e.g., sitagliptin, saxagliptin):

○ **Efficacy:** Increases insulin secretion and reduces glucagon levels.
○ **Safety Profile:** Generally well-tolerated, with a low risk of hypoglycemia.
○ **Considerations:** No specific interactions with sickle cell disease medications reported.

GLP-1 Receptor Agonists (e.g., liraglutide, dulaglutide):

○ **Efficacy:** Enhances insulin secretion and reduces appetite.
○ **Safety Profile:** May cause nausea and gastrointestinal side effects.
○ **Considerations:** No direct interactions with sickle cell disease medications reported. Monitor for gastrointestinal side effects.

## 8. Pain management of DM in SCA

Managing pain in individuals with both diabetes mellitus (DM) and sickle cell anemia (SCA) requires a comprehensive approach that addresses the unique challenges and complexities of both conditions.^[64]^ Pain management strategies need to be tailored to accommodate the chronic pain associated with SCA while considering the implications of pain medications on diabetes management. Recognize and differentiate the types of pain experienced by individuals with DM and SCA.^[85]^ Diabetes-related pain often includes neuropathic pain (tingling, burning, or shooting pain due to nerve damage), while sickle cell pain is typically episodic and related to vaso-occlusive crises. Engage a multidisciplinary team that includes pain specialists, hematologists, endocrinologists, and other relevant healthcare professionals to develop a comprehensive pain management plan.

Implement nonpharmacological strategies to manage pain, such as physical therapy, occupational therapy, acupuncture, massage therapy, cognitive-behavioral therapy (CBT), relaxation techniques, and mindfulness-based practices.^[86]^ These techniques can help reduce pain perception and improve overall well-being without relying solely on medications. Select pain medications judiciously, considering their effectiveness, safety, and potential interactions with diabetes medications. Be cautious with opioid medications due to the risk of addiction, tolerance, and adverse effects. nonopioid analgesics and adjuvant medications may be considered as alternatives or adjuncts to manage pain. Develop individualized pain management plans that address the specific needs and preferences of the individual while considering the complexities of managing both DM and SCA. Tailor the treatment plan based on the type, severity, and frequency of pain episodes.^[63]^ Monitor individuals closely for any side effects or interactions between pain medications and medications used to manage diabetes or sickle cell disease. Adjustments may be needed to minimize adverse effects while effectively managing pain. Emphasize the importance of lifestyle modifications, including a balanced diet, regular physical activity, stress management, and adequate hydration, which can contribute to pain management and overall well-being. Provide comprehensive education to individuals and caregivers about pain management strategies, including the use of medications, nonpharmacological approaches, and self-management techniques. Schedule regular follow-up visits to assess the effectiveness of pain management strategies, make necessary adjustments, and ensure ongoing support for pain relief. Address the emotional and psychological aspects of living with chronic pain in individuals with both conditions. Provide counseling, support groups, and resources to help individuals cope with the challenges.^[87]^ Managing pain in individuals with both DM and SCA requires a holistic and individualized approach that considers the complexities of both conditions. Close collaboration among healthcare providers and active involvement of the individual are essential for developing effective pain management strategies while optimizing overall health outcomes.

## 9. Prevention and monitoring of complications of DM in SCA

Managing diabetes mellitus (DM) in the presence of sickle cell anemia (SCA) requires a proactive approach to prevent complications and closely monitor for potential adverse effects of both conditions.^[88]^ The coexistence of DM and SCA presents unique challenges due to the potential interactions and overlapping complications associated with each condition. Schedule regular medical checkups with healthcare providers specializing in diabetes and sickle cell disease.^[89]^ These checkups should include comprehensive assessments of blood sugar levels, HbA1c, hemoglobin levels, kidney function, eye examinations, and assessment of any signs of sickle cell-related complications. Encourage and support healthy lifestyle habits, including a balanced diet, regular physical activity, adequate hydration, stress management, and avoidance of harmful habits like smoking and excessive alcohol consumption. These lifestyle changes can help in managing both conditions and reducing the risk of associated complications. Emphasize the importance of tight blood sugar control in diabetes management.^[90]^ Regular monitoring of blood glucose levels, adherence to diabetes management plans, and medications are crucial to prevent diabetes-related complications. Ensure that individuals with both DM and SCA receive recommended vaccinations, including pneumococcal and influenza vaccines.^[91]^ Vaccinations are essential for preventing infections, especially in individuals with functional asplenia due to SCA. Ensure strict adherence to prescribed medications for both conditions. This includes diabetes medications, medications to manage sickle cell complications (such as hydroxyurea), and any other prescribed treatments to prevent complications.

## 10. Monitoring for complications

Regularly monitor blood sugar levels using appropriate methods, such as frequent fingerstick measurements or continuous glucose monitoring (CGM).^[92]^ This monitoring helps in tracking blood sugar fluctuations and adjusting diabetes management strategies accordingly. Monitor hemoglobin levels, hematocrit, and other hematologic parameters to assess the impact of sickle cell disease on red blood cells, detect any abnormalities, and manage potential complications, such as anemia or vaso-occlusive crises.^[93]^ Periodically assess kidney function through tests like serum creatinine, estimated glomerular filtration rate (eGFR), and urine albumin-to-creatinine ratio (ACR) to detect early signs of diabetic nephropathy or kidney impairment due to sickle cell disease.^[94]^ Table 1 shows medications in sickle cell anemia patients with diabetes, Table 2 shows challenges in managing diabetes mellitus patients with sickle cell anemia, Table 3 shows therapeutic approaches for glycemic control in patients with diabetes mellitus and sickle cell anemia, Table 4 shows management of vaso-occlusive crises in patients with diabetes mellitus and sickle cell anemia, Table 5 shows renal complications management in patients with diabetes mellitus and sickle cell anemia and Table 6 shows complications and considerations in long-term management (authors provided).

**Table 2:** Challenges in managing diabetes mellitus patients with sickle cell anemia.

Challenge	Description
Hyperglycemia	Sickle cell anemia can cause chronic inflammation, leading to insulin resistance and difficulty in glycemic control.
Hypoglycemia	Increased risk of hypoglycemia due to irregular food intake and unpredictable glucose metabolism in sickle cell crisis.
Vaso-occlusive crises	Episodes of vaso-occlusion can lead to tissue ischemia, exacerbating existing complications of diabetes.
Chronic pain management	Balancing pain control with glucose management can be challenging due to the need for opioid analgesics in sickle cell patients.
Renal complications	Both sickle cell anemia and diabetes can independently contribute to renal dysfunction, increasing the risk of complications.

**Table 3:** Therapeutic approaches for glycemic control in patients with diabetes mellitus and sickle cell anemia.

Therapeutic approach	Description
Individualized treatment plans	Tailoring diabetes management plans to accommodate the unique needs and challenges of sickle cell patients.
Continuous glucose monitoring (CGM)	Utilizing CGM to provide real-time glucose data and adjust therapy accordingly, especially during sickle cell crises.
Insulin therapy	Adjusting insulin regimens to accommodate fluctuations in insulin sensitivity during sickle cell crises and hyperglycemic episodes.
Lifestyle modifications	Encouraging regular exercise, healthy eating habits, and stress management to improve overall health and glycemic control.
Patient education and support	Providing comprehensive education on self-management strategies and regular monitoring to empower patients in their care.

**Table 4:** Management of vaso-occlusive crises in patients with diabetes mellitus and sickle cell anemia.

Intervention	Description
Pain management	Utilizing analgesics, including opioids, NSAIDs, and nonpharmacological interventions, to alleviate pain during crises.
Intravenous fluids	Hydration therapy to prevent dehydration and promote blood flow, reducing the risk of vaso-occlusion.
Oxygen therapy	Supplemental oxygen to improve tissue oxygenation and mitigate the effects of hypoxia during sickle cell crises.
Blood transfusion	Exchange transfusion or packed red blood cell transfusion may be necessary to reduce sickle hemoglobin levels and alleviate symptoms.
Hydroxyurea therapy	Long-term use of hydroxyurea to reduce the frequency and severity of vaso-occlusive crises in sickle cell patients.

**Table 5:** Renal complications management in patients with diabetes mellitus and sickle cell anemia.

Intervention	Description
Blood pressure control	Strict blood pressure control through pharmacotherapy and lifestyle modifications to prevent or slow the progression of diabetic nephropathy and sickle cell nephropathy.
Renal function monitoring	Regular monitoring of renal function through laboratory tests (e.g., serum creatinine, estimated glomerular filtration rate) to detect early signs of kidney damage.
ACE inhibitors/ARBs	Renin-angiotensin system inhibitors may be used to manage hypertension and reduce proteinuria in patients with renal complications.
Diabetic nephropathy management	Optimal glycemic control, lipid management, and lifestyle modifications to prevent or delay the progression of diabetic kidney disease.
Sickle cell nephropathy management	Aggressive hydration, avoidance of nephrotoxic medications, and treatment of urinary tract infections to minimize the risk of sickle cell nephropathy.

**Table 6:** Complications and considerations in long-term management.

Complication/consideration	Description
Peripheral neuropathy	Monitoring for and managing diabetic neuropathy, which may be exacerbated by sickle cell anemia-related vaso-occlusive episodes.
Foot care	Regular foot exams and preventive measures to mitigate the risk of foot ulcers and amputations in diabetic patients with sickle cell anemia.
Retinopathy	Ophthalmologic evaluations and prompt treatment of diabetic retinopathy to preserve vision in patients with concurrent sickle cell anemia.
Cardiopulmonary complications	Regular cardiovascular assessments and pulmonary function tests to detect and manage complications such as pulmonary hypertension and cardiac dysfunction.
Psychosocial support	Providing psychological support and resources to help patients cope with the challenges of managing 2 chronic conditions simultaneously.

Encourage a healthy diet, regular physical activity, and weight management. Regular monitoring of blood glucose levels is essential to adjust treatment plans as needed. Individualize medication regimens based on factors such as age, comorbidities, and patient preferences. Metformin is often recommended as the initial pharmacologic therapy for type 2 diabetes, unless contraindicated. Consider insulin therapy for those with type 2 diabetes not achieving glycemic targets with oral medications. Recommend additional medications based on individual needs, including SGLT-2 inhibitors, DPP-4 inhibitors, GLP-1 receptor agonists, and others. Manage hypertension and dyslipidemia to reduce cardiovascular risk. Tailor treatment plans to the individual’s needs, considering factors like age, life expectancy, comorbidities, and hypoglycemia risk.^[95–102]^

## 11. Conclusion

Managing diabetes mellitus (DM) in patients with sickle cell anemia (SCA) presents a complex set of challenges, necessitating a thoughtful and comprehensive approach to care. The interplay between these 2 chronic conditions can complicate both disease management and significantly impact the patient’s quality of life. However, with careful consideration and a multidisciplinary approach, healthcare providers can address these challenges effectively. Therapeutic approaches must be tailored to the unique needs and circumstances of each patient, taking into account their medical history, pain crises, medication interactions, nutritional restrictions, and psychological well-being. Close monitoring of blood glucose levels is essential, as is the development of individualized treatment plans that balance the control of diabetes with the management of SCA.

It is crucial for healthcare providers to be aware of the potential complications and increased risks associated with these conditions, and to educate patients and their families about the importance of self-care, regular medical follow-up, and the impact of lifestyle choices on their health. While managing DM in SCA patients may present significant challenges, with a patient-centered and multidisciplinary approach, these challenges can be addressed, and individuals can achieve better control of their health and well-being.

## Author contributions

**Conceptualization:** Emmanuel Ifeanyi Obeagu.

**Methodology:** Emmanuel Ifeanyi Obeagu, Getrude Uzoma Obeagu.

**Supervision:** Emmanuel Ifeanyi Obeagu.

**Validation:** Emmanuel Ifeanyi Obeagu.

**Visualization:** Emmanuel Ifeanyi Obeagu.

**Writing – original draft:** Emmanuel Ifeanyi Obeagu, Getrude Uzoma Obeagu.

**Writing – review & editing:** Emmanuel Ifeanyi Obeagu, Getrude Uzoma Obeagu.
